# Deviation from power law of the global seismic moment distribution

**DOI:** 10.1038/srep40045

**Published:** 2017-01-05

**Authors:** Isabel Serra, Álvaro Corral

**Affiliations:** 1 Centre de Recerca Matemàtica, Edifici C, Campus Bellaterra, E-08193 Barcelona, Spain; 2 Departament de Matemàtiques, Facultat de Ciències, Universitat Autònoma de Barcelona, E-08193 Barcelona, Spain

## Abstract

The distribution of seismic moment is of capital interest to evaluate earthquake hazard, in particular regarding the most extreme events. We make use of likelihood-ratio tests to compare the simple Gutenberg-Richter power-law (PL) distribution with two statistical models that incorporate an exponential tail, the so-called tapered Gutenberg-Richter (Tap) and the truncated gamma, when fitted to the global CMT earthquake catalog. Although the Tap distribution does not introduce any significant improvement of fit respect the PL, the truncated gamma does. Simulated samples of this distribution, with parameters *β* = 0.68 and *m*_*c*_ = 9.15 and reshuffled in order to mimic the time occurrence of the order statistics of the empirical data, are able to explain the temporal heterogeneity of global seismicity both before and after the great Sumatra-Andaman earthquake of 2004.

The Gutenberg-Richter (GR) law is not only of fundamental importance in statistical seismology^1^ but also a cornerstone of non-linear geophysics^2^ and complex-systems science^3^. It simply states that, for a given region, the magnitudes of earthquakes follow an exponential probability distribution. As the (scalar) seismic moment is an exponential function of magnitude, when the GR law is expressed in terms of the former variable, it translates into a power-law distribution^4^,^5^, i.e.,


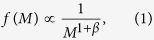


with *M* seismic moment, *f*(*M*) its probability density, (fulfilling 
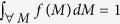
), the sign “∝” denoting proportionality, and the exponent 1 + *β* taking values close to 1.65. This simple description provides rather good fits of available data in many cases^6^,^7^,^8^,^9^, with, remarkably, only one free parameter, *β*. A totally equivalent characterization of the distribution uses the survivor function (or complementary cumulative distribution), defined as


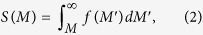


for which the GR power law takes the form *S*(*M*) ∝ 1/*M*^*β*^.

The power-law distribution has important physical implications, as it suggests an origin from a critical branching process or a self-organized-critical state^3^,^10^,^11^. Nevertheless, it presents also some conceptual difficulties, due to the fact that the mean value 〈*M*〉 provided by the distribution turns out to be infinite^4^,^12^. These elementary considerations imply that the GR law cannot be naively extended to arbitrarily large values of *M*, and one needs to introduce additional parameters to describe the tail of the distribution, coming presumably from finite-size effects. However, a big problem is that the change from power law to a faster decay seems to take place at the highest values of *M* that have been observed, for which the statistics are very poor^13^.

Kagan^7^ has enumerated the requirements that an extension of the GR law should fulfil; in particular, he considered, among other: (i) the so called tapered (Tap) Gutenberg-Richter distribution (also called Kagan distribution^14^), with a survivor function given by


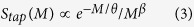


and (ii) the (left-) truncated gamma (TrG) distribution, for which the density is


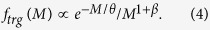


Note that both expressions have essentially the same functional form, but the former refers to the survivor function and the later to the density. As *f*(*M*) = −*dS*(*M*)/*dM*, differentiation of *S*_*tap*_(*M*) in (i) shows the difference between both distributions. In both cases, parameter *θ* represents a crossover value of seismic moment, signalling a transition from power law to exponential decay; so, *θ* gives the scale of the finite-size effects on the seismic moment. The corresponding value of (moment) magnitude (sometimes called corner magnitude) can be obtained from 
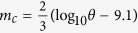
, when the seismic moment is measured in N · m^15^,^16^.

Kagan^7^ also argues that available seismic catalogs do not allow the reliable estimation of *θ*, except in the global case (or for large subsets of this case), in particular, he recommends the use of the centroid moment tensor (CMT) catalog^17^,^18^. From his analysis of global seismicity, and comparing the values of the likelihoods, Kagan^7^ concludes that the tapered GR distribution gives a slightly better fit than the truncated gamma distribution, for which in addition the estimation procedure is more involving. In any case, the *β*−value seems to be universal (at variance with *θ*), see also refs 9, 19 and 20.

Nevertheless, the data analyzed by Kagan^7^, from 1977 to 1999, comprises a period of relatively low global seismic activity, with no event above magnitude 8.5; in contrast, the period 1950–1965 witnessed 7 of such events^21^. Starting with the great Sumatra-Andaman earthquake of 2004, and following since then with 5 more earthquakes with *m* ≥ 8.5 (up to the time of submitting this article), the current period seems to correspond to the past higher levels of activity.

Main *et al*.^22^ and Bell *et al*.^23^ have re-examined the problem of the seismic moment distribution including recent global data (shallow events only). Using a Bayesian information criterion (BIC), Bell *et al*.^23^ compare the plain GR power law with the tapered GR distribution, and conclude that, although the tapered GR gives a significantly better fit before the 2004 Sumatra event, the occurrence of this changes the balance of the BIC statistics, making the GR power law more suitable; that is, the power law is more parsimonious, or simply, is enough for describing global shallow seismicity when the recent mega-earthquakes are included in the data. Similar results have been published in ref. 24.

In the present paper we revisit the problem with more recent data, including also the truncated gamma distribution, using other statistical tools, and reaching somewhat different conclusions: when data includes periods of high seismic activity, indeed, the tapered GR distribution does not introduce any significant improvement with respect to the power law^23^, but the truncated gamma does.

## Data, Models and Maximum Likelihood Estimation

As Main *et al*.^22^ and Bell *et al*.^23^, we analyze the global CMT catalog^17^,^18^, in our case for the period between January 1, 1977 and October 31, 2013, with the values of the seismic moment converted into N · m (1 dyn · cm = 10^−7^ N · m). We restrict to shallow events (depth <70 km) and, in order to avoid incompleteness, to magnitude *m* > 5.75 (equivalent to *M* > 5.3 · 10^17^ N · m), as Main *et al*.^22^ and Bell *et al*.^23^. This yields 6150 events.

As statistical tools, we use maximum likelihood estimation (MLE) for fitting, and likelihood-ratio (LR) tests for comparison of different fits. Maximum likelihood estimation is the best-accepted method in order to fit probability distributions, as it yields estimators which are invariant under re-parameterizations, and which are asymptotically efficient for regular models, in particular for exponential families^25^ (the three models under consideration here are regular, and the PL and the TrG belong to the exponential family). When maximum likelihood is used under a wrong model, what one finds is the closest model to the true distribution in terms of the Kullback-Leibler divergence^25^.

Model selection tests based on the likelihood ratio have the advantage that the ratio is invariant with respect to changes of variables (if these are one-to-one^25^). Moreover, for comparing the fit of models in pairs, LR test is preferable in front of the computation of differences in BIC or AIC (Akaike information criterion), as the test relies on the fact that the distribution of the LR is known, under a suitable null hypothesis, which provides a significance level (or level of risk) to its value. So, LR tests constitute probability-based model selection (in contrast to BIC and AIC). But note that the log-likelihood-ratio is equal to the difference of BIC or AIC when the number of parameters of the two models is the same.

In order to perform MLE it is necessary to specify the densities of the distributions, including the normalization factors. In our case, all distributions are defined for *M* above the completeness threshold *a*, i.e., for *M* > *a*, being zero otherwise (as mentioned above, *a* is fixed to 5.3 × 10^17^ N · m). For the power-law (PL) distribution (which yields the GR law for the distribution of *M*) Eq. (1) reads


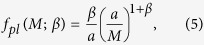


with *β* > 0. For the tapered Gutenberg-Richter,


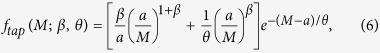


with *β* > 0 and *θ* > 0. And for the left-truncated (and extended to *β* > 0) gamma distribution;


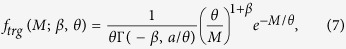


with −∞ < *β* < ∞ and *θ* > 0, and with 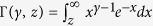 the upper incomplete gamma function, defined for *z* > 0 when *γ* < 0.

We summarize the parameterization of the densities as *f*(*M*; Θ), where Θ = {*β, θ*} for the Tap and TrG distributions and Θ = *β* for the power law. Note that for the TrG distribution, it is clear that the exponent *β* is a shape parameter and *θ* is a scale parameter; in fact, these parameters play the same role in the Tap distribution, which turns out to be a mixture of two truncated gamma distributions, one with shape parameter *β* and the other with *β* − 1, but with common scale parameter *θ*. Exactly,


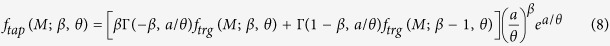


(in our case, the contribution of the second TrG will be only about 0.14%). In contrast, the power law lacks a scale parameter. In all cases the completeness threshold *a* is a truncation parameter, but it is kept fixed and is not a free parameter, therefore.

Other authors consider the upper truncated power-law distribution^13^,^26^, given by *f*(*M*; *β, θ*) ∝ 1/*M*^1+*β*^ for *a* < *M* < *θ*, and zero otherwise; then *θ* becomes a truncation parameter. We disregard this model because such an abrupt truncation is unphysical^7^, because the occurrence of one single earthquake with size larger than the resulting value of *θ* invalidates the selected model, and because the fact that the support of the distribution involves the unknown parameter *θ* leads to a violation of the regularity conditions for which standard likelihood theory holds^25^.

The knowledge of the probability densities allows the direct computation of the likelihood function as 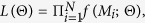 where *M*_*i*_ are the *N* observational values of the seismic moment. Maximization of the likelihood function with respect the values of the parameters leads to the maximum-likelihood estimation 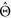 of these parameters, with 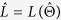 the value of the likelihood at its maximum. Note that the independence assumption that is implicit in the expression for *L*(Θ) arises in fact as the maximum-entropy solution when there is no information about dependence^27^. If the data cannot be considered independent, the MLE results will just describe a marginal distribution *f*(*M*; Θ) of the sample under consideration, and inference about the underlying population will not be possible, as the sample may be not representative of the population. In any case, the results of MLE for our three models are reported in Table 1, and an illustration of the corresponding fits is provided in the Supplementary Information (SI). Although the TrG model has the highest likelihood one has to perform a proper model comparison.

## Model Comparison

A powerful method for comparison of pairs of models is the likelihood-ratio test, specially suitable when one model is nested within the other, which means that the first model is obtained as a special case of the second one. This is the case of the power-law distribution with respect to the other two distributions; indeed, the power law is nested both within the Tap and within the truncated gamma, as taking *θ* → ∞ in any of the two leads to the power-law distribution. This is easily seen taking into account that *S*_*tap*_(*M*) = (*a*/*M*)^*β*^*e*^−(*M−a*)/*θ*^, or just performing the limit in the expression for *f*_*tap*_(*M*) above. For the truncated gamma distribution, when doing the *θ* → ∞ limit in *f*_*trg*_(*M*) one needs to use that, for *γ* < 0, *z*^*γ*^/Γ(*γ, z*) → −*γ* when *z* → 0, see ref. 28 for *γ* ≠ −1, −2, …

Given two probability distributions, 1 and 2, with 1 nested within 2, the likelihood-ratio test evaluates 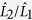, where 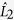 is the likelihood (at maximum) of the “bigger” or “full” model (either Tap or TrG) and 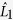 corresponds to the nested or null model (power law in our case). Taking logarithms we get the log-likelihood-ratio


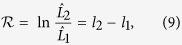


with 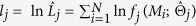, where *f*_*i*_ denotes the probability density function of the distribution *j* for every *j* = 1, 2, and the MLE corresponds to 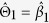 and 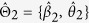. In order to compare the fit provided by the two distributions, it is necessary to characterize the distribution of 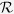.

Let *n*_1_ and *n*_2_ be the number of free parameters in the models 1 and 2, respectively. In general, if the models are nested, and under the null hypothesis that the data comes from the simpler model, the probability distribution of the statistic 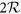 in the limit *N* → ∞ is a chi-squared distribution with degrees of freedom equal to *n*_2_ − *n*_1_ > 0. So, for *n*_2_ = *n*_1_ + 1,


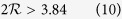


with a level of risk equal to 0.05. Note that the chi-squared distribution provides a penalty for model complexity as the “range” or “scale” of the distribution is given directly by the number of the degrees of freedom. This likelihood-ratio test constitutes the best option to choose among models 1 and 2, in the sense that it has a convergence to its asymptotic distribution faster than any other test^29^. The null and alternative hypotheses correspond to accept model 1 or 2, respectively, although the acceptance of model 1 does not imply the rejection of 2, it is simply that the “full” model 2 does not bring any significant improvement with respect the simpler model 1, which is more parsimonious.

On the other hand, when the nesting of distribution 1 within 2 takes place in such a way that the space of parameters of the former one lies within a boundary of the space of parameters of distribution 2, the approach just explained for the asymptotic distribution of 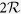 is not valid^30^,^31^. This happens when testing both the Tap or the TrG distributions in front of the power-law distribution, as the *θ* →∞ limit of the latter corresponds to the boundary of the parameter space of the two other distributions, and then, what one should obtain for 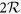 is a mixture of a chi-square and a Dirac delta function. Nevertheless, this latter result is also unapplicable in our case, as the power-law distribution does not fulfil the sufficient conditions stated in ref. 30, due to the divergence of the second moment^32^. This illustrates part of the difficulties of performing proper model selection when fractal-like distributions are involved^33^. In order to obtain the distribution of 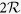 and from there the *p*−values of the LR tests, we are left to the simulation of the null hypothesis. We advance that the results seem to indicate that the distribution of 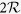, for high percentiles, is close to chi-square with one degree of freedom, so that Eq. (10) is approximately valid, but we lack a theoretical support for this fact.

**Figure 1:**
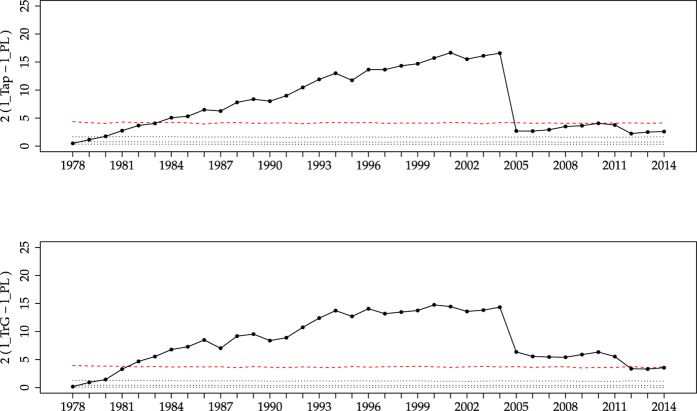
Results of the likelihood-ratio tests. The points (joined by lines) denote the value of the statistic 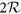 for the empirical data. Lines show different percentiles of the distribution of 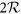 for 10000 simulations of the power-law null hypothesis with the same number of data (dotted black lines: first, second, and third quartiles), including the critical value of the test (at level 0.05, dashed red line). The abscissa corresponds to the ending point of a time window starting always in Jan 1, 1977. Note that the year is considered a continuous variable (not a categorical variable), so, the time window ending on Dec 31, 2004 takes value 2004.99…≃2005. (**a**) Tap distribution versus power law. (**b**) Truncated gamma versus power law.

Let us proceed, using this method, by comparing the performance of the power-law and Tap fits when applied to the global shallow seismic activity, for time windows starting always in 1977 and ending in the successive times indexed by the abscissa in Fig. 1(a) (as in ref. 23). The log-likelihood-ratio of these fits (times 2), is shown in the figure together with the critical region of the test. In agreement with Bell *et al*.^23^, we find that: (i) the power-law fit can be safely rejected in front of the Tap distribution for any time window ending between 1984 and before 2004; and (ii) the results change drastically after the occurrence of the great 2004 Sumatra earthquake, for which the power law cannot be rejected at the 0.05 level. So, for parsimony reasons, the power law becomes preferable in front of the Tap distribution for time windows ending later than 2004. The fact that, for these time windows, the Tap distribution cannot be distinguished from the power law is also in agreement with previous results showing that the contour lines in the likelihood maps of the Tap distribution are highly non-symmetric and may be unbounded for smaller levels of risk^7^,^24^,^34^.

When we compare the power-law fit with the truncated gamma, using the same test, for the same data, the results are more significant, see Fig. 1(b). The situation previous to 2004 is nearly the same, with an extremely poor performance of the power law; but after 2004, despite a big jump again in the value of the likelihood ratio, the power law remains non-acceptable, at the 0.05 level. It is only after the great Tohoku earthquake of 2011 that the *p*−value of the test enters slightly into the non-rejection region, but keeping values very close to the 0.05 limit. From here we conclude that, in order to find an alternative to the power-law distribution, the truncated gamma distribution is a better option than the Tap distribution, as it is more clearly distinguishable from the power law (for this particular data).

At this point, a direct comparison between these two distributions (Tap and TrG) seems pertinent. In this case we may use the likelihood-ratio test of Vuong for non-nested models^35^,^36^. As the number of parameters is the same for both models, their log-likelihood-ratio coincides with the difference in BIC or AIC, but the LR procedure incorporates a statistical test which specifies the distribution of the statistic under consideration. Unfortunately, the results are inconclusive, as no significance difference shows up. This is not surprising if one considers that the LR test for non-nested models is less powerful than the LR test for nested models used above.

In order to check the possible influence of the different heterogeneous populations present in global seismicity, associated to different tectonic zones, we have separately analyzed subduction zones, similarly as done in ref. 37, using Flinn-Engdahl’s regionalization^6^. The results for the LR tests are qualitatively the same, with the main difference that the values of *l*_*trg*_ − *l*_*pl*_ become somewhat smaller (not shown); nevertheless, as long as a time window of several years is considered, the power-law hypothesis can always be rejected except after the Tohoku earthquake. The resulting MLE parameters for the TrG are 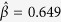 and 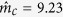 (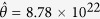 N · m) for *N* = 4067 events. Then, the slightly larger value of 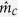 with respect the global case (Table 1) makes the power law a bit harder to reject.

## Simulated Data with Temporal Reshuffling

As we have seen, in contrast to the Tap, the TrG distribution does bring an improvement with respect the PL, so, we concentrate on further comparisions between TrG and PL. With the purpose of gaining further insight, we simulate random samples following the truncated gamma distribution, with the parameters 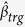 and 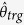 obtained from MLE of the complete dataset (Table 1), with the same truncation parameter *a* and number of points (*N* = 6150) also. To avoid that the conclusions depend on the time correlation of magnitudes in the empirical data, we reshuffle the simulated data in such a way that the temporal occurrence of the order statistics in the seismic moment is the same as for the empirical data; in other words, the largest simulated event is assigned to take place at the time of the 2011 Tohoku earthquake (the largest of the CMT catalog^23^), the second largest at the time of the 2004 Sumatra event, and so on. In this way, we model earthquake seismic moments as arising from a gamma distribution with fixed parameters, with occurrence times given by the empirical times, and with practically the same seismic-moment correlations as the empirical data.

**Figure 2:**
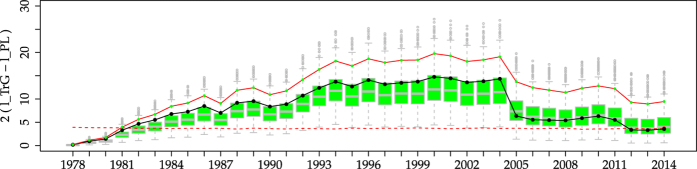
Comparison of the empirical values of the statistic 2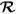 = 2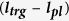 (points with lines, shown also in Fig. 1) with those resulting from 1000 simulations of the TrG distribution (boxplots) using the final parameters of Table 1 (i.e., *β* = 0.681 and *m*_*c*_ = 9.15). The 95th percentile of the boxplots is also shown, in continuous red. Simulated seismic moments are reshuffled as explained in the text to make the comparison possible. The agreement between empirical data and simulations is very remarkable. The red dashed line is the same as in Fig. 1. Remember that the central lines in the boxplots represent the three quartiles of the distribution of 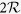.

We simulate 1000 datasets with *N* = 6150 each. The results, summarized in Fig. 2 using boxplots^38^, show that the behaviour of the empirical data is not atypical in comparison with this gamma modelling. In nearly all time windows the empirical data lies in between the first and third quartile of the simulated data, although before 2004 the empirical values are close to the third quartile whereas after 2004 they lay just below the median. This leads us to compute the statistics of the jump in the log-likelihood-ratio between 2004 and 2005. The estimated probability of having a jump larger than the empirical value is around 4.5%, which is not far from what one could accept from the gamma modelling explained above. Thus, a TrG distribution, with fixed parameters, is able to reproduce the empirical findings, if the peculiar time ordering of magnitude of the real events is taken into account. Notice also that, although the simulated data come from a TrG distribution, they are not distinguishable from a power law for about half of the simulations of the last time windows, as the critical region is close to the median indicated by the boxplots.

**Figure 3:**
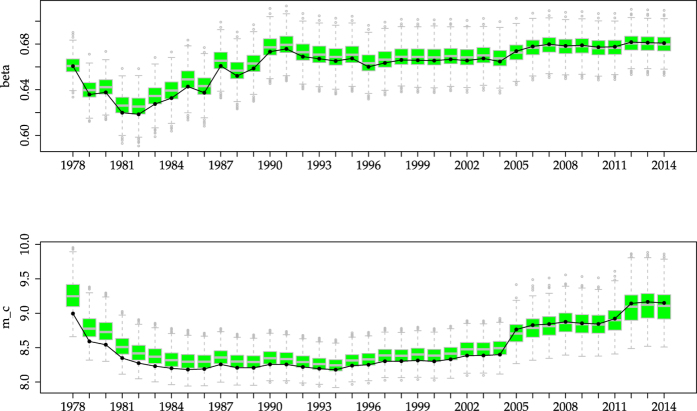
Comparison between empirical estimated parameters 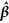 and 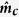 for the TrG distribution (points with lines) and the estimations for the 1000 simulations of Fig. 2 (i.e., TrG with *β* = 0.681 and *m*_*c*_ = 9.15 with temporal reshuffling, boxplots). The different stability of both parameters is apparent, as well as the similarity between data and simulations. (**a**) 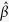. (**b**) 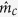.

We can also compare the evolution of the estimated parameters for the empirical dataset and for the reshuffled TrG simulations, with a good agreement again, see Fig. 3. There, it is clear that although the exponent *β* reaches very stable values relatively soon (around 1990), the scale parameter *θ* (equivalent to *m*_*c*_) is largely unstable, and the occurrence of the biggest events makes its value increase.

**Figure 4:**
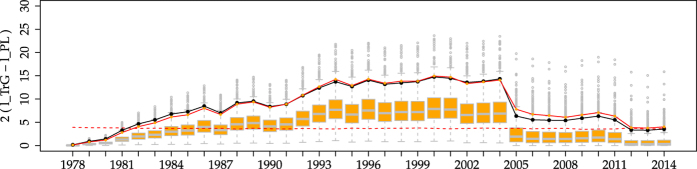
As Fig. 2, but simulating a power law with parameter *β* = 0.685 (Table 1) instead of a TrG distribution. The reshuffling is also as in Fig. 2, as explained in the text. The simulations lead, on average, to values of the likelihood ratio smaller than the empirical ones. Note that the difference with Fig. 1(b) is that there (i) there is no reshuffling and (ii) the value of *β* in the simulations corresponds to the obtained 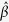 for each time window.

As a complementary control we invert the situation, simulating 1000 synthetic power-law datasets with *β* = 0.685 (Table 1), *a* = 5.3 × 10^17^ N · m, and *N* = 6150, for which the same time reshuffling is performed, in such a way that the order of the order statistics is the same. In this case, the results of the simulations lead, on average, to much smaller values of the log-ratio in comparison with the empirical data, which corresponds to the limit of rejection for many time windows, see Fig. 4. So, a power-law distribution with temporal reshuffling cannot account for the empirical results as clearly as a truncated gamma distribution. Doing the same with a Tap distribution one finds something in between, see SI.

## Discussion

Testing different statistical models for the distribution of seismic moment of global shallow seismicity (using the CMT catalog) we have found that, in contrast to the Tap distribution, the truncated gamma brings significant improvement with respect to the power law. Moreover, in order to reproduce the time evolution of the statistical results, it suffices that independent seismic moments following a truncated gamma distribution with fixed parameters *β* = 0.68 and *m*_*c*_ = 9.15 are reshuffled so that the peculiar empirical time sequence of magnitudes is maintained (note that after reshuffling independence is broken). So, despite the fact that the future occurrence of more and larger mega-earthquakes could significantly change the value of parameter *m*_*c*_^13^, the current value is enough to explain the available data. Although ref. 13 claims that no less than 45,000 events are necessary for the reliable estimation of *m*_*c*_, our simulations with 6150 events indicate otherwise, see for instance the last boxplot for the estimation of *m*_*c*_ in Fig. 3, which yields a mean value of 9.11, with a standard deviation of 0.24, totally consistent with the results in Table 1. We conclude that the fundamental problem in the estimation of *m*_*c*_ is not the number of available data but the temporal heterogeneity of the seismic moment distribution. We have also found, with a similar reshuffling procedure, that a power-law distribution cannot account for the empirical findings. Direct comparison of Figs 2 and 4 shows how the TrG distribution outperforms the power law. Additionally, it would be very interesting to investigate if the high values of the likelihood ratio attained before the 2004 Sumatra event could be employed to detect the end of periods of low global seismic activity. Certainly, more case studies would be necessary for that purpose.

As extra arguments in favour of the truncated gamma distribution in front of the tapered GR, we can bring not statistical evidence but physical plausibility and statistical optimality. On the one hand, the former distribution can be justified as coming from a branching process that is slightly below its critical point^12^,^39^. Further reasons that may support the truncated gamma are that this arises (i) as the maximum entropy outcome under the constrains of fixed (arithmetic) mean and fixed geometric mean of the seismic moment^40^; (ii) as the closest to the power law, in terms of the Kullback-Leibler divergence, when the mean seismic moment is fixed^41^; and (iii) as a stable distribution under a fragmentation process with a power-law transition rate^41^. We are not aware of similar theoretical support in favour of the Tap distribution. On the other hand, it is straightforward to check that the truncated gamma belongs to the exponential family^25^, in contrast to the Tap distribution. And it is well known that estimators in the exponential family achieve the Cramér-Rao lower bound for any sample size, in contrast to other regular models, where the bound is only achieved asymptotically.

## Additional Information

**How to cite this article**: Serra, I. and Corral, Á. Deviation from power law of the global seismic moment distribution. *Sci. Rep.* **7**, 40045; doi: 10.1038/srep40045 (2017).

**Publisher's note:** Springer Nature remains neutral with regard to jurisdictional claims in published maps and institutional affiliations.

## Supplementary Material

Supplementary Information

## Figures and Tables

**Table 1:** Maximum likelihood estimation of the parameters with their standard errors (s.e.) and maximum value of the log-likelihood function, *l* = ln 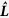 when the PL, Tap, and TrG distributions are fitted to the seismic moment of shallow CMT earthquakes, using the whole data set (*N* = 6150).

		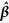	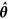 (N · m)	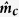	*l* (*M* in N · m)	*l* − *l*_*pl*_	*l*_*trg*_ − *l*_*tap*_
PL	MLE	0.685	∞	∞	−268466.609		
	s.e.	0.009					
Tap	MLE	0.684	3.3 × 10^22^	8.94	−268465.315	1.294	
	s.e.	0.009	2.6 × 10^22^	0.23			
TrG	MLE	0.681	6.7 × 10^22^	9.15	−268464.844	1.765	0.471
	s.e.	0.009	6.6 × 10^22^	0.27			

The standard error for 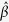 and 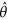 is computed from the Fisher information matrix and corresponds to one standard deviation of the distribution of each parameter. The standard error for 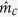 is computed from that of 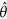 using the delta method^42^.
