# Supplementary material for: Deviation from power law of the global seismic moment distribution
Source: Sci Rep. 2017 Jan 5;7:40045. doi: 10.1038/srep40045 (PMC5215546; doi:10.1038/srep40045)
Supplement: Supplementary Information [file srep40045-s1.pdf]

Supplementary Information for
Deviation from power law of the global seismic moment distribution

Isabel Serra[1,*] and Álvaro Corral[1,2,*]

[1]Centre de Recerca Matemàtica, Edifici C, Campus Bellaterra, E-08193 Barcelona, Spain.
[2]Departament de Matemàtiques, Facultat de Ciències, Universitat Autònoma de Barcelona, E-08193 Barcelona, Spain,
[*]iserra@crm.cat,acorral@crm.cat

ABSTRACT

We provide complementary figures to the main text.

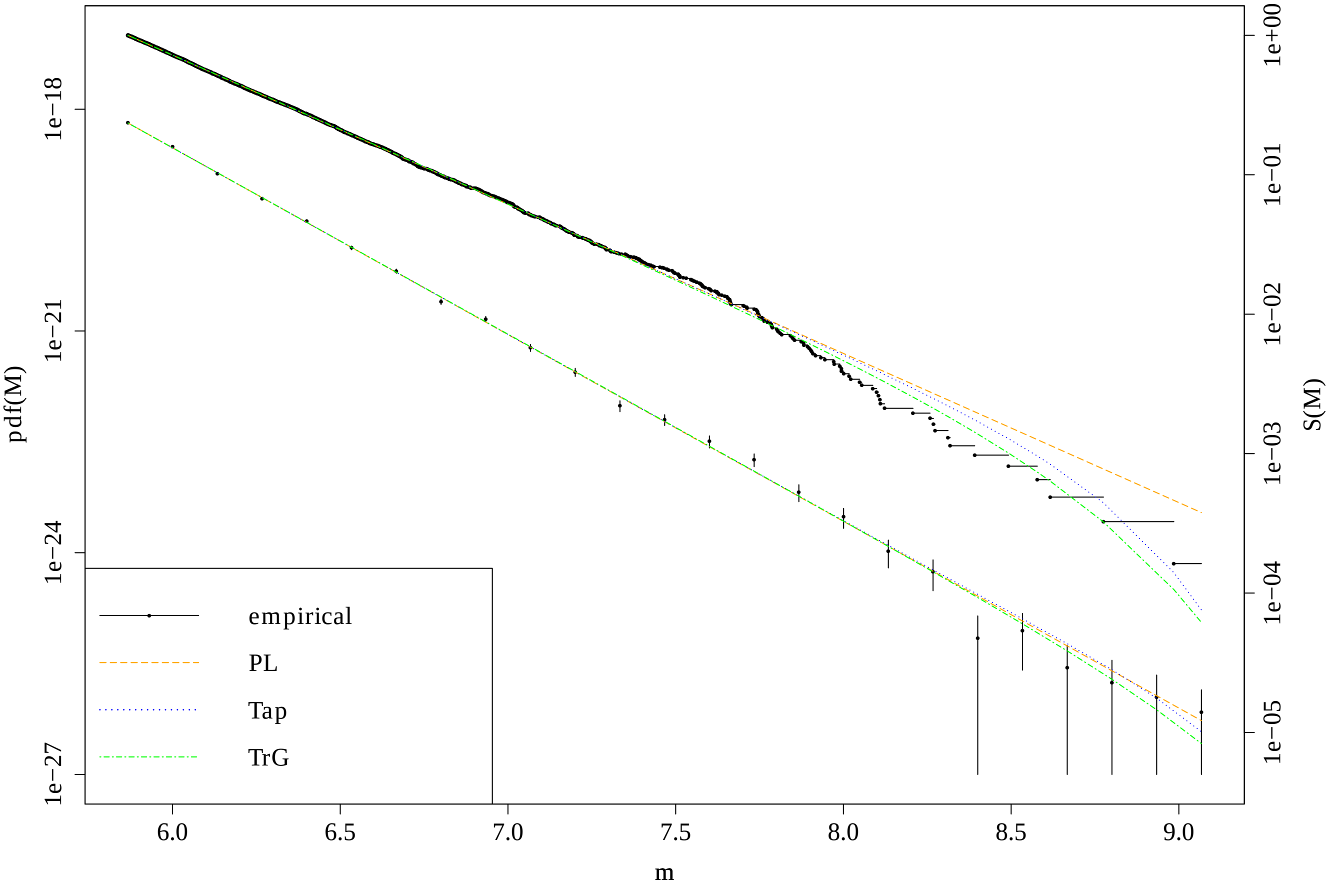

Supplementary Figure S 1. Comparison of the fits of the three models (PL, Tap, TrG), using the complementary cumulative distribution function (upper curves, right axis) and the probability density function of the seismic moment (pdf, lower curves, left axis). m in the $x-$axis is the moment magnitude corresponding to seismic moment M. The empirical data correspond to earthquakes in the CMT catalog, as explained in the main text; the parameters of the fits are those in Table 1. Units of the pdf are $\mathrm{N}^{-1}\cdot\mathrm{m}^{-1}$. The empirical density is estimated using the method of Ref. [8] (with the error bars arising from one standard deviation in the number of counts).

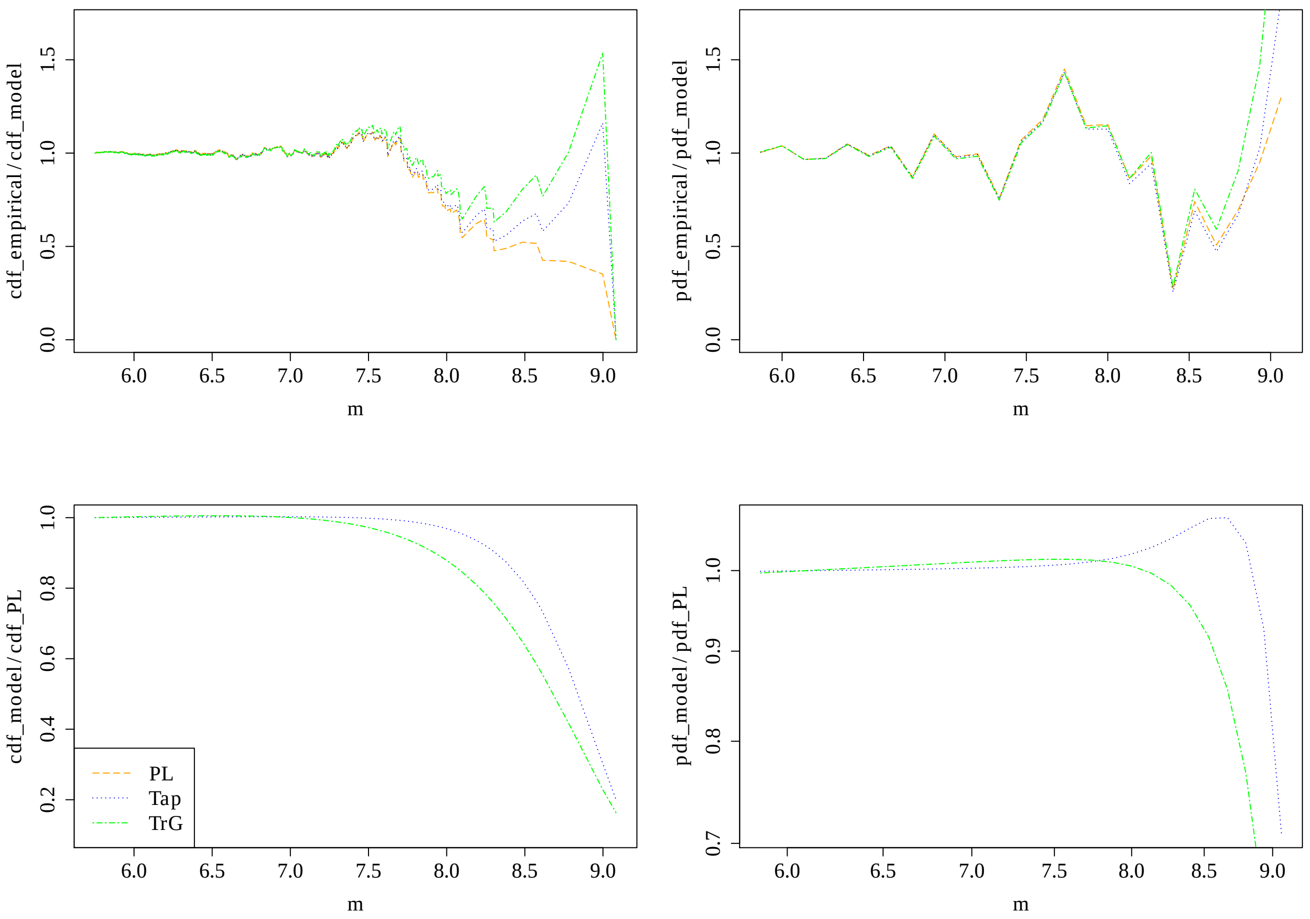

Supplementary Figure S 2. Same data as in Fig. S 1. Top figures: Comparison of the three fits (PL, Tap, TrG), using the ratio between the empirical distribution and the fitted one (left: complementary cumulative distribution function, cdf; right: probability density of seismic moment). Bottom figures: Comparison of fitted models. Ratio between Tap and PL (labelled Tap) and between TrG and PL (labelled TrG) (left: cdf's; right: pdf's). Notice the relative bump for the Tap pdf.

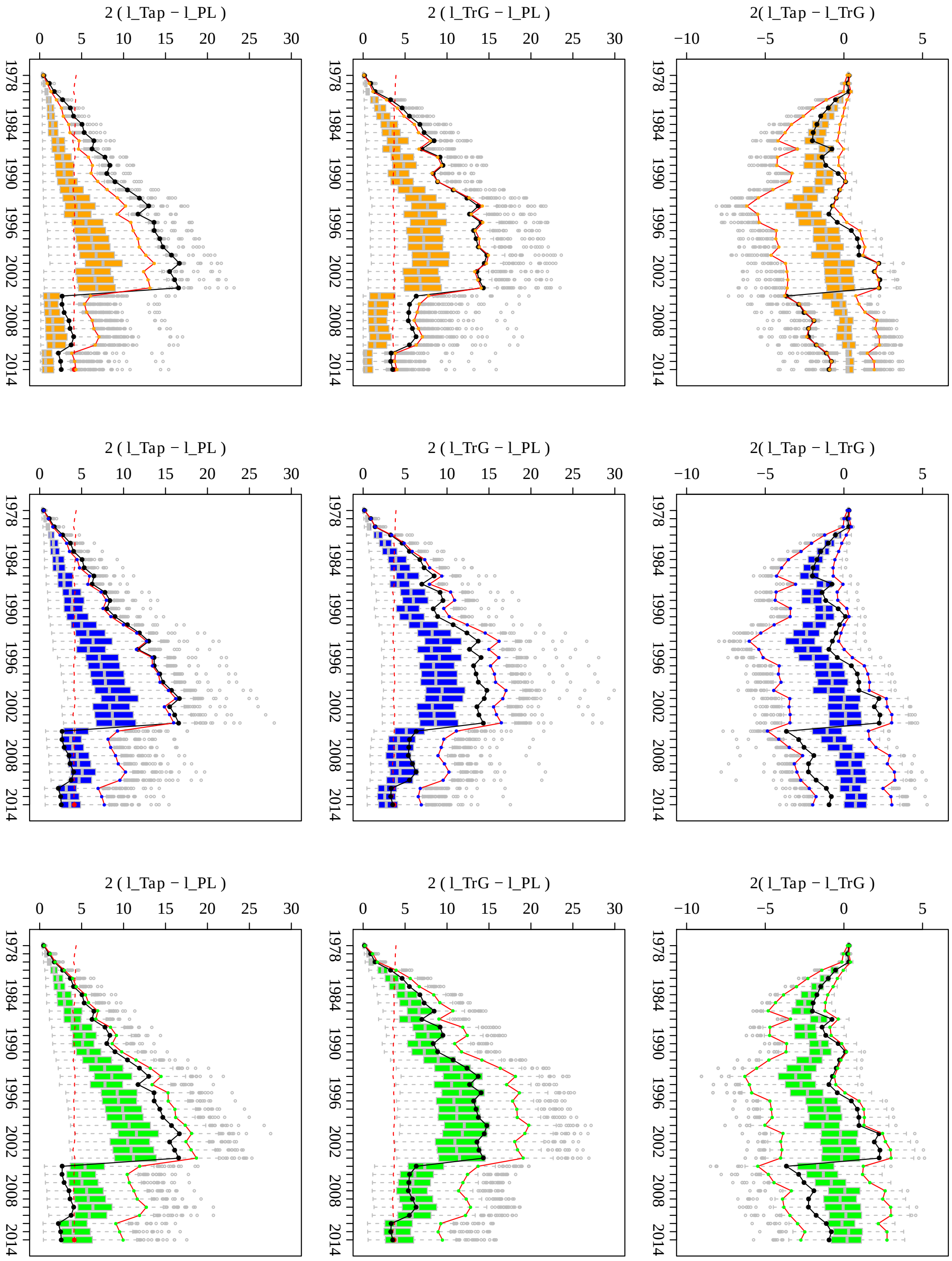

Supplementary Figure S 3. Comparison of the empirical values of the statistic $2(l_{m'} - l_m)$ (black points with lines) with the values resulting from 1000 simulations of a null distribution (boxplots), using the final parameters of Table 1 and temporal reshuffling. m and m' label the models whose log-likelihood l is computed, and the null hypothesis corresponds to PL (1st horizontal column, boxplots in orange), Tap (2nd column, in blue), and TrG (3rd column, in green). The critical region with 95 % confidence level of the boxplots is also shown, in continuous red. The red dashed line is as in Fig. 1 of the main text. Notice that Figs. 2 and 4 there are included in the central vertical file of the present figure (last and first, respectively). The equivalent of Fig. 2 for the Tap distribution would be the one in the middle of the left vertical file ($l_{tap} - l_{pl}$ with simulated Tap events, in blue). The conclusion is that the TrG gamma distribution outperforms the other two, in the sense that it cannot be rejected in any case.
